# Metabolic Investigation of *Phelipanche aegyptiaca* Reveals Significant Changes during Developmental Stages and in Its Different Organs

**DOI:** 10.3389/fpls.2017.00491

**Published:** 2017-04-07

**Authors:** Noam Nativ, Yael Hacham, Joseph Hershenhorn, Evgenia Dor, Rachel Amir

**Affiliations:** ^1^Migal Galilee Technology CenterKiryat Shmona, Israel; ^2^Biotechnology Department, Tel-Hai CollegeUpper Galilee, Israel; ^3^Weed Research Department, Newe Ya'ar Research CenterRamat-Yishay, Israel

**Keywords:** developmental stages, GS-MS analysis, parasite's organs, *Phelipanche aegyptiaca*, primary metabolic profiling, total nitrogen

## Abstract

*Phelipanche aegyptiaca* Pers. is a root holoparasitic plant considered to be among the most destructive agricultural weeds worldwide. In order to gain more knowledge about the metabolic profile of the parasite during its developmental stages, we carried out primary metabolic and lipid profiling using GC-MS analysis. In addition, the levels of amino acids that incorporate into proteins, total protein in the albumin fraction, nitrogen, reduced sugars, and phenols were determined. For the assays, the whole plants from the four developmental stages—tubercle, pre-emergent shoot, post-emergent shoot, and mature flowering plants—were taken. Thirty-five metabolites out of 66 differed significantly between the various developmental stages. The results have shown that the first three developmental stages were distinguished in their profiles, but the latter two did not differ from the mature stage. Yet, 46% of the metabolites detected did not change significantly during the developmental stages. This is unlike other studies of non-parasitic plants showing that their metabolic levels tend to alter significantly during development. This implies that the parasite can control the levels of these metabolites. We further studied the metabolic nature of five organs (adventitious roots, lower and upper shoot, floral buds, and flowers) in mature plants. Similar to non-parasitic plants, the parasite exhibited significant differences between the vegetative and reproductive organs. Compared to other organs, floral buds had higher levels of free amino acids and total nitrogen, whereas flowers accumulated higher levels of simple sugars such as sucrose, and the putative precursors for nectar synthesis, color, and volatiles. This suggests that the reproductive organs have the ability to accumulate metabolites that are required for the production of seeds and as a source of energy for the reproductive processes. The data contribute to our knowledge about the metabolic behavior of parasites that rely on their host for its basic nutrients.

## Introduction

*Orobanche* and *Phelipanche* spp. are obligate plant-parasitic plants in the Orobanchaceae family (Joel et al., 2007). Due to their achlorophyllous nature, they are constrained to obtain their nutritional resources by feeding off broad-leaf plants using the haustorium, a unique organ in parasitic plants that links the parasite to the root of the hosts. Through the haustorium, the parasite diverts water, and nutrients from the host (Westwood, 2013), leading to severe yield loss, and quality in numerous vegetables crops (Joel et al., 2007; Pérez-de Luquea et al., 2010; Fernández-Aparicio et al., 2016). It was suggested that once the connection between the parasite and the host roots is established, the parasite functions as an active sink, redirecting solutes away from autotrophic sink tissues and subsequently leading to a decrease in the accumulation of host biomass (Péron et al., 2016).

Despite accumulated knowledge about the parasite's agricultural damage, lifecycle and mode of action, our knowledge about the primary metabolic profiling and its metabolism is mostly unknown. Several studies previously measured the levels of a few amino acids, sugars, or polyols in several parasitic plants (e.g., Aber et al., 1983; Abbes et al., 2009a,b; Delavault, 2015), however, they usually concentrated on one group of metabolites, or even on one metabolite. Metabolic profiling or metabolomics is a rapidly developing technology of the post-genomics era that focuses on global changes in biological samples and enables determining metabolic differences in plants during various developmental stages, growth conditions and stresses (Nakabayashi and Saito, 2015).

We recently carried out a primary metabolic profiling analysis of the tips of young shoots of *Phelipanche aegyptiaca* Pers. that emerged from the “spiders” stage. In addition, the parasite's infected tomato roots and non-parasitized roots were analyzed (Hacham et al., 2016). Major differences in metabolites contents between the parasite and the tomato roots were defined, suggesting that *P. aegyptiaca* has its own metabolism that differs significantly in its regulation from those found in their host. These findings support previous lines of evidence showing significant accumulations of metabolites such as mannitol in the parasite (Delavault et al., 2002). In addition to primary metabolites, we also determined the total phenolic compounds that mostly belong to secondary metabolites, revealing that their levels were about three-fold higher compared to the host-infected roots (Hacham et al., 2016). The results have also shown that the levels of most of the metabolites in the infected roots were similar to the levels detected in the non-infected roots, except for seven metabolites whose levels increased in the infected vs. the non-infected roots (Hacham et al., 2016).

The motivation of the current study is to enhance our knowledge about the *P. aegyptiaca* primary metabolic profiling. To achieve this, we defined the changes in the whole plant of *P. aegyptiaca* during four distinct developmental stages and in five different organs of the mature and flowering plant. Such data could not only contribute to our knowledge about the behavior of parasitic plants and their metabolic nature, but also provide more data about the similarity to non-parasitic plants.

## Materials and methods

### Plant material

Tomato (*Solanum lycopersicon*) cultivar M-82 seeds were obtained from Tarsis Agricultural Chemicals Ltd., Israel. Seeds were collected from inflorescences of *P. aegyptiaca* parasitizing tomato grown on Kibbutz Beit Ha'shita in the 2015 season. Seeds were stored and held in the dark at 4°C until use. M82 tomato seedlings were planted in 2-L pots (Tefen Nachsholim, Israel) using medium-heavy clay-loam soil containing a dry weight basis of 55% clay, 23% silt, 20% sand, 2% organic matter, pH 7.1 (one plant per pot). Slow-release fertilizer at a concentration of 0.6% (w/v) (Osmocote, ScottsMiracle-Gro, Marysville, OH) and *P. aegyptiaca* seeds at a concentration of 15 ppm (15 mg seeds kg−^1^ soil ~2,250 seeds kg−^1^) were added to the soil. The above-mentioned components were mixed to homogeneity in a cement mixer for 10 min. The pots were placed in a net house and drip-irrigated as needed.

**Figure 1:**
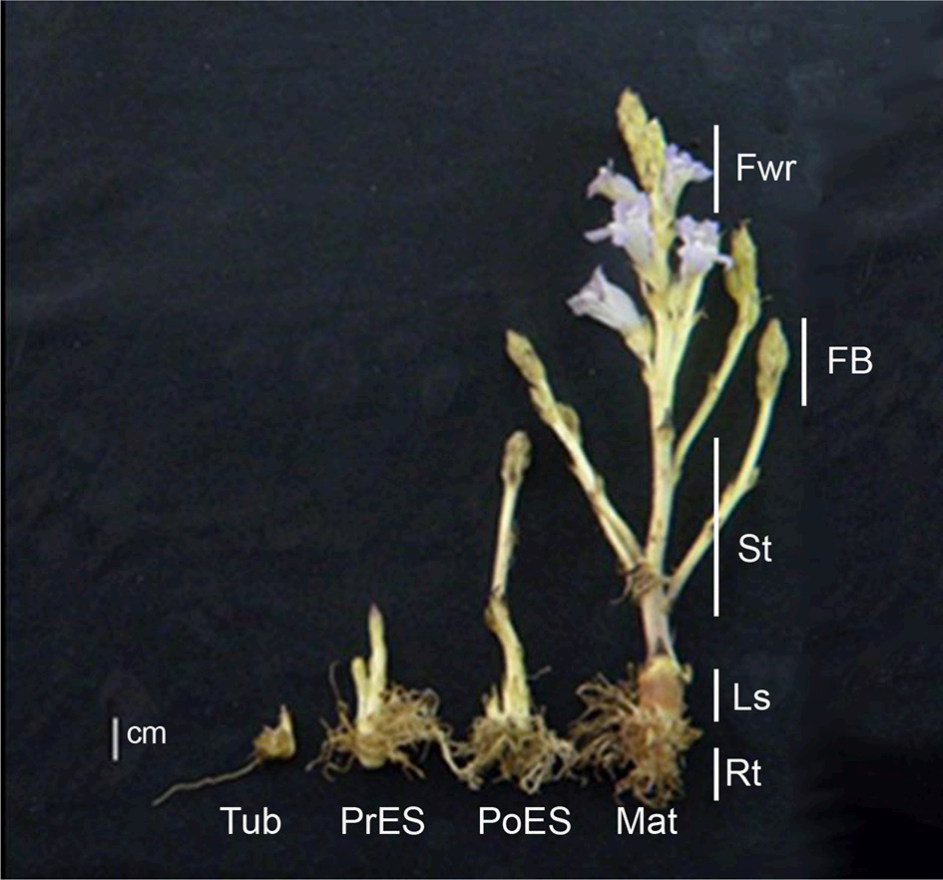
**Comparative illustration of the four key life stages and five organs collected from mature flowering plants of ***Phelipanche aegyptiaca*****. The developmental stages are: Tub, Tubercle; PeES, Pre-emergent shoot; PoES, Post-emergent shoot; and Mat, Mature flowering plant. The different organs are: Rt, Adventitious roots; LS, underground pre-emergent shoot; St, post-emergent shoot; FB, Floral buds; and Fwr, Flowers. Samples were collected 12 weeks after planting.

The different developmental stages and different organs were collected 12 weeks after planting. Four different developmental stages were collected (Figure 1): Tubercle carrying adventitious roots; pre-emergent shoots that also include adventitious roots; post-emergent vegetative shoots that also include very small and condense floral buds; and whole mature *P. aegyptiaca* that have adventitious roots, pre- and post-emergent shoots and reproductive tissue, including floral buds and flowers (according to Westwood et al., 2012). Five organs were collected from mature adult plants that have all the organs: Adventitious roots; underground pre-emergent shoot; post-emergent stem; floral buds; and flowers (Figure 1). The whole parasitic plants during the four developmental stages and the five organs were frozen in liquid nitrogen and dried by lyophilization. The samples were then ground to dried powder using TissueLyser (Qiagen Retsch MM301) for the various analyses.

### Extraction and analysis of primary metabolites using gas chromatography-mass spectrometry (GC-MS)

Primary metabolites were extracted from 20 mg *P. aegyptiaca* dried powder in 1 mL of pre-cooled extraction buffer containing HPLC-grade methanol:chloroform:H_2_O (2.5:1:1 v/v/v) and transferred into new 2 mL lock-cap Eppendorf tubes. Each sample was added with 4.6 μL of norleucine internal standard (2 mg mL^−1^ in HPLC-grade H_2_O), vortexed for 10 s, and centrifuged for 10 min at 20,817 g at 4°C pre-cooled table-top centrifuge to permit the separation of polar (methanol/water) and non-polar (chloroform) phases. The methanol-water fractions were treated and derivatized as previously described (Matityahu et al., 2013; Cohen et al., 2014). The single-ion mass method was used for soluble and protein-incorporated amino acid determination with the RXI-5-Sil MS capillary column (RESTEK; 30 m, 0.25-mm i.d., and 0.25-mm thickness), while the total-ion-count method was used for metabolic profiling and separation using the VF-5 ms capillary column (Agilent; 30 m + 10 m EZ-guard, 0.25-mm i.d., and 0.25-mm thicknesses). All analyses were carried out on a GC-MS system (Agilent 7890A) coupled with a mass selective detector (Agilent 5975c) and a Gerstel multipurpose sampler MPS2 (Cohen et al., 2014). Peak finding, peak integration and retention time correction were performed with the Agilent GC/MSD Productivity ChemStation package (http://www.agilent.com). Areas of the peaks were normalized to integral standard (norleucine) signal. The identification of these metabolites was based on standards (amino acids, fatty acids, and most of the primary metabolites), and the spectra of all the peaks were compared with commercially available electron mass spectrum libraries, NIST, and WILEY.

### Extraction of total amino acids

For total amino acid determination including protein-bound amino acids, 20 mg of *P. aegyptiaca* dried powder from different developmental stages and plant organs were mixed with distilled water. After two centrifugation cycles (14,000 rpm for 20 min), 25 μl of protein extraction were placed in a glass tube with norleucine solution (60 μg/ml). The samples were frozen at −70°C and lyophilized, followed by 6N HCl acidic hydrolysis for 22 h at 110°C under vacuum (Frank et al., 2015). Hydrolysis products were suspended in 0.45 ml DDW and 0.3 ml chloroform. Following centrifugation (14,000 rpm, 20 min), 0.3 ml aliquots from the upper phase were collected, and samples were silylated, and analyzed as previously described (Matityahu et al., 2013).

### Determination of fatty acid contents using GC-MS

Fatty acid composition was analyzed using an established GC-MS protocol (Bai et al., 2012). Three hundred microliters from the non-polar chloroform phases taken from the samples prepared for metabolic profiling were collected and transferred into new Eppendorf tubes. To each sample, 7 μL of heptadecanoic acid (C17:0) internal standard (5 mg mL^−1^ in HPLC-grade chloroform) was added. The chloroform phase was dried under a stream of nitrogen gas, and lipids were transmethylated with 2% (v/v) H_2_SO_4_ in methanol at 85°C for 1 h. The reaction was terminated by the addition of water, and fatty acid methyl esters (FAMEs) were extracted in hexane and transferred to 300 μL autosampler vials. FAMEs were quantified and identified on a GC-MS system (Agilent 7890A) coupled with a mass selective detector (Agilent 5975c) and a Gerstel multipurpose sampler MPS2. Helium was used as the carrier gas at a flow rate of 0.87531 mL/min. A 1 μL sample was injected into the split mode, the inlet temperature was 250°C and the pressure was 15.0 psi. Separation was achieved on a 70% cyanopropyl polysilphenylene-siloxane column (BPX70, SGE Analytical Science, 25 m, 0.22 mm, 0.25 μm) using the temperature gradient from 130°C (hold time 1 min) to 210°C (linear increase of 3°C min^−1^; hold time 5 min) and finally to 240°C (linear increase of 10°C min^−1^; hold time 2 min). FAMEs were identified by co-chromatography with authentic standards (Sigma-Aldrich).

### Total protein and nitrogen determinations

For total protein determination, 20 mg of *P. aegyptiaca* dried powder of tissues from the samples were ground in 400 μL buffer phosphate pH = 7.8 with a protease inhibitor cocktail (Sigma, P9599). After two centrifugation cycles (14,000 rpm for 20 min), total protein was determined using a Bradford reagent (Bio-Rad Hercules, Calif.) in three sample concentrations (Bradford, 1976). Bovine serum albumin was used as standard.

For total nitrogen measurement, 20 mg dried powder of the *P. aegyptiaca* tissues was digested with 0.4 mL of H_2_SO_4_ overnight. Afterwards 2 mL of H_2_O_2_ were added for 24 min at a heating block at 280°C. Samples were then cooled for 15 min at room temperature and left overnight in order to enable the evaporation of H_2_O_2_. Double-distilled water (DDW) was added to bring the total volume to 100 mL. The solution was diluted 10 times and used to determine the concentration of total N (Wolf, 1982; Prodhan et al., 2016). The total dissolved nitrogen was determined using a total organic N analyzer (Multi NC 2100S, Analytik Jena, Jena, Germany).

### Total reducing sugars determination

Reducing sugars were measured colorimetrically using the Sumner method following carbohydrates and starch hydrolysis (Sumner, 1924). Forty mg dry weights of each of the *P. aegyptiaca* samples from the different developmental stages and different organs were ground using a Restch MM 301 homogenizer and extracted in 40 ml HCl 1N at 70°C for 2 h. After cooling, the pH was adjusted to 7.3 and the volume to 50 ml with water. One half ml of the samples was then mixed with 1.5 ml water and 2 ml of Sumner reagent and left for 5 min at 90°C. After cooling, the reducing sugars were detected at 550 nm by the UV-vis spectrophotometer 1240 (Shimadzu) (Hacham et al., 2016). A standard curve was created using glucose in a range of 50–500 μg.

### Total phenolic compounds content determination

For total phenolic compounds content determination, most of which are secondary metabolites, 20 mg dried powder from samples of *P. aegyptiaca* were ground in 0.5 ml water, and the colorimetric method, which modified the Ben Nasr method for small volumes (Ben Nasr et al., 1996), was employed. Ten microliters of the extraction sample were loaded on a 96-well ELISA plate. To each well, 50 μl of 10% Folin-Ciocalteu reagent and 40 μl of 7.5% (w/v) Na_2_CO_3_ were added. The plate was incubated for 40 min at 37°C and then read at 765 nm (Infinite M200PRO Tecan, Grodig Austria). A standard curve was created using gallic acid in a range of 0.5–10 μg.

### Statistical analyses

For the developmental stages, 5–10 parasitic plants were taken for each biological replicate, while for the different organs at least five plants for each biological replicate were taken. The data represent the mean of four independent replications. Statistical significance was evaluated using JMP software version 8.0 (SAS Institute Inc., Cary, NC). Significant differences between treatments were calculated according to the Turkey-Kramer HSD test (*p* < 0.05). Principal component analysis (PCA) and a heat-map of GC-MS data were conducted using the MetaboAnalyst 3.0 comprehensive tool (http://metaboanalyst.ca/; Xia et al., 2015) with Auto scaling (mean-centered and divided by the standard deviation of each variable) manipulations. Graphs were compiled using GraphPad Prism 5.01 scientific software (http://www.graphpad.com/).

## Results

### Metabolic profile of *P. aegyptiaca* during four developmental stages

Four different developmental stages were taken from *P. aegyptiaca* for primary metabolic profiling analyses: Tubercle; pre-emergent shoot; post-emergent vegetative shoots; and mature flowering plants (Figure 1). The whole plants from each developmental stage were ground and used for global primary metabolic profile analyses, as well as a lipid profile analysis, using established GC-MS protocols (Bai et al., 2012; Cohen et al., 2014; Hacham et al., 2016). The analysis revealed 66 annotated metabolites having a significantly higher signal-to-noise value. The annotated metabolites belong to seven distinct biochemical groups: Amino acids (19); sugars (16 in total, 13 annotated and 3 non-annotated); polyols (6); tricarboxylic acid cycle (TCA) intermediates (4); organic acids (5); sugar acids (5); fatty acids (6); and others (5) (Supplemental Table S1).

**Figure 2:**
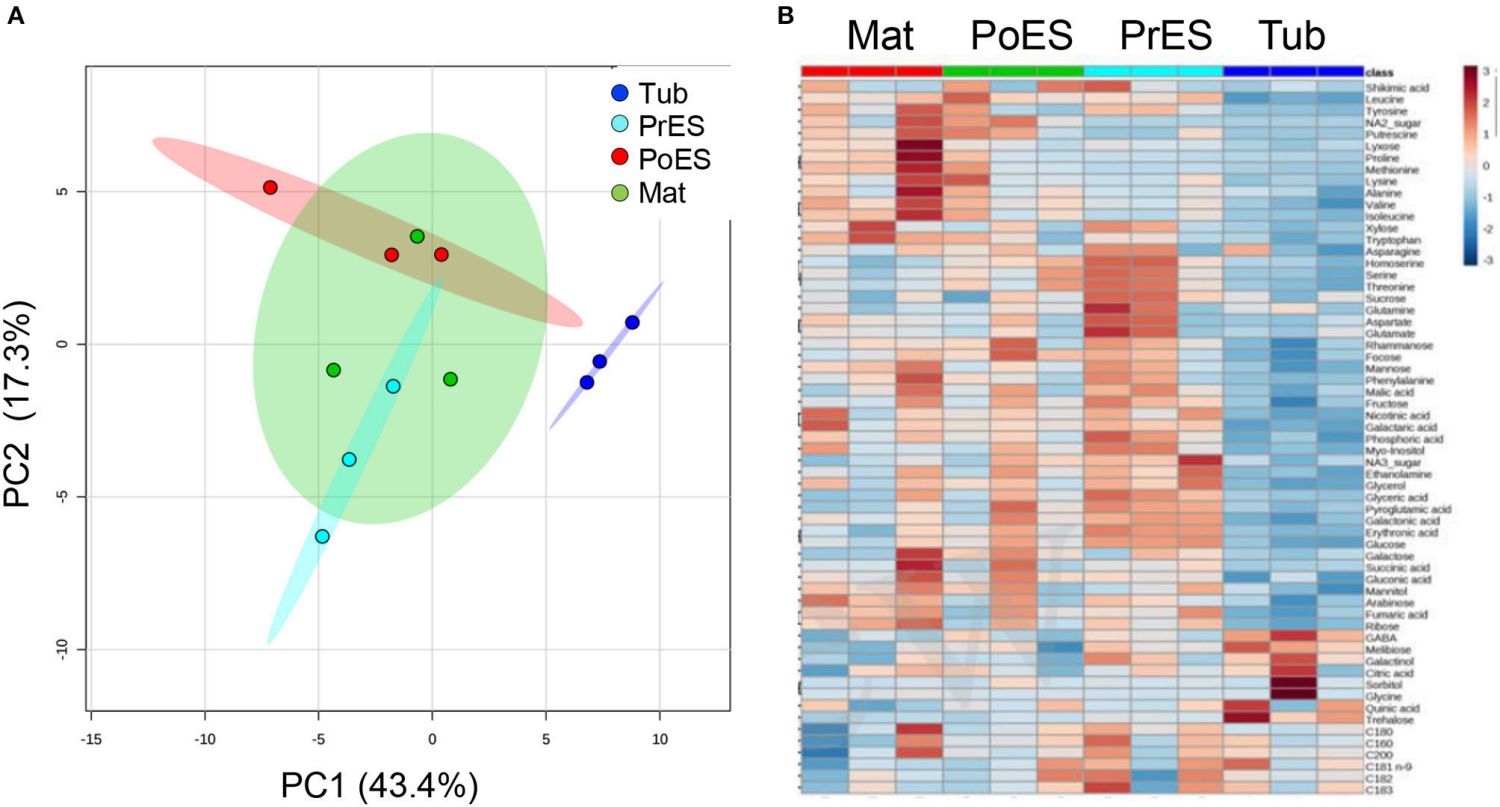
**Graphical representation of changes in ***Phelipanche aegyptiaca*** metabolite profiles at four developmental stages. (A)** Principal component analyses (PCA) applied to the four developmental stages according to their entire primary metabolome set of 66 metabolites. The data points are displayed as projections onto the two primary axes (eigenvectors). Variances explained by the first two components (PC1 and PC2) appear in parentheses; **(B)** heat-map of these 66 primary metabolites detected by GC-MS. The data represent three replicates for each developmental stage.

The metabolite profiles of these four developmental stages were plotted onto a PCA (Xia et al., 2015). The two principal components account for 65% of the total variance (Figure 2A). The PCA analysis showed that the first three developmental stages—tubercle, pre-emergent shoot, and post-emergent vegetative shoot—were separated from one another. However, the pre-emergent shoot and post-emergent shoot stages were not separated from the mature stage (Figure 2A). A heat-map analysis showed that compared to the other developmental stages, the tubercle has the lowest contents of most of the metabolites detected (Figure 2B; Supplemental Table S1).

**Figure 3:**
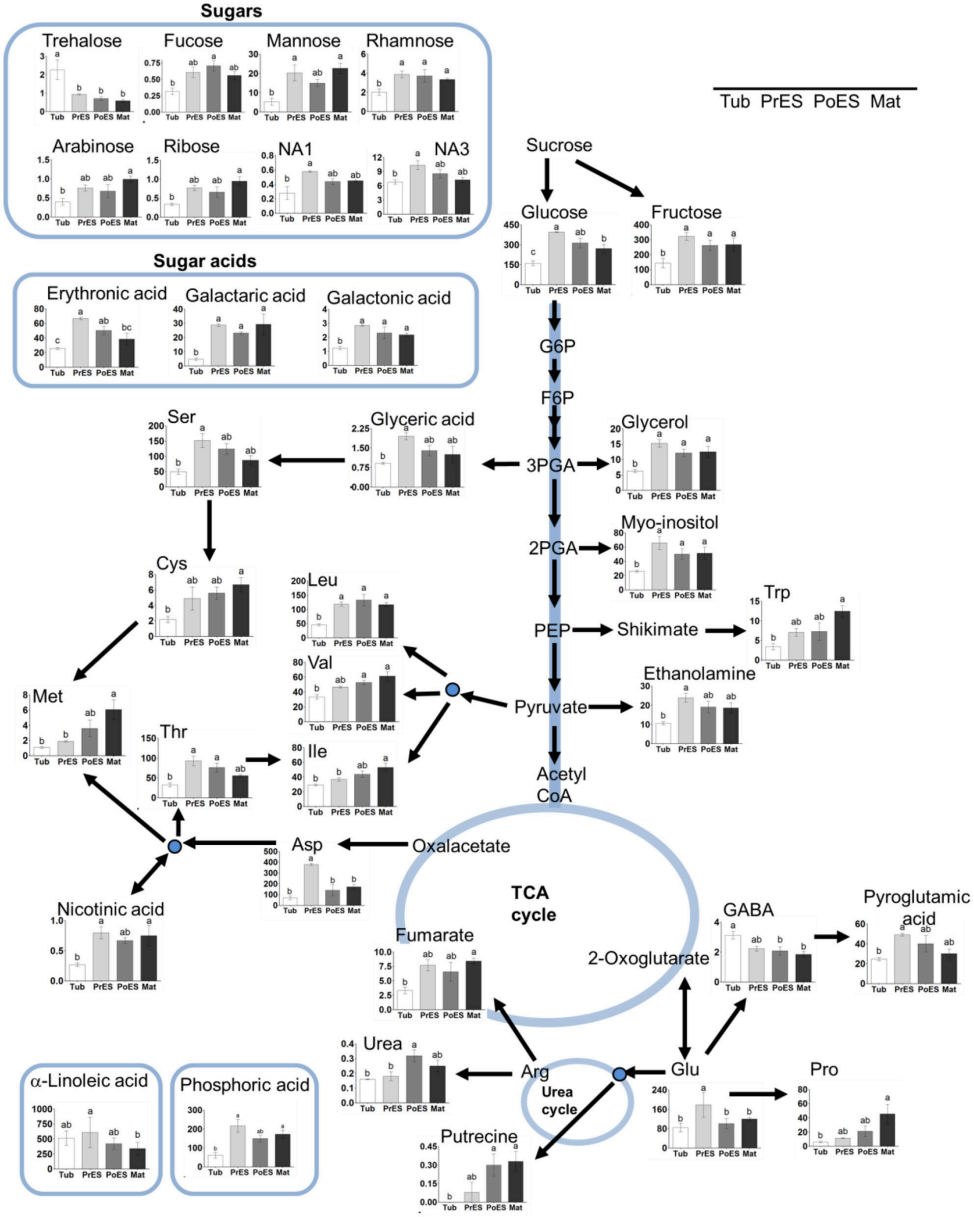
**The levels of primary metabolites and lipids of ***Phelipanche aegyptiaca*** during four developmental stages**. Only metabolites whose levels changed significantly in at least one developmental stage were presented. The developmental stages are: Tub, Tubercle; PeES, Pre-emergent shoot; PoES, Post-emergent shoot; and Mat, Mature flowering plant. The y axis of amino acids is shown in nmol/g DW, for fatty acid in μg/g DW, while for the other metabolites it represents the area of relative m/z response of each metabolite following normalization to the norleucine internal standard. Data shown are means ± *SE* of three replicates for each type of developmental stage. Significance was calculated according to the Turkey-Kramer HSD test (*p* < 0.05) and is identified by different small letters. G6P, glucose 6-phosphate; F6P, fructose 6-phosphate; 3-PGA, 3-phosphoglyceric acid; 2-PGA, 2-phosphoglyceric acid; PEP, phosphoenolpyruvic acid; Glu (glutamic acid); Gln (glutamine); Asp, aspartic acid; Ser, serine; Ile, isoleucine; Val, valine; Leu, leucine; Trp, tryptophan; Cys, cysteine; Met, methionine; Thr, threonine; Glu, glutamic acid; Pro, proline; GABA, γ-aminobutyric acid.

To gain a better resolution, each metabolite whose level changed significantly between the different stages was plotted separately on Figure 3. Examination of the tubercle metabolites revealed that trehalose showed significantly higher level at this stage compared to the other developmental stages (Figure 3). In addition, the level of γ-aminobutyric acid (GABA) was high at this stage compared to the last two developmental stages (Figure 3). The metabolic profile of the different developmental stages basically shows that the levels of nine metabolites gradually increased during the four developmental stages. These are methionine, isoleucine, valine, tryptophan, cysteine, proline, arabinose, ribose, and fumaric acid (Figure 3; Supplemental Table S1). In the pre-emergent shoots, only glutamate and aspartate were significantly higher compared to the other stages, however, the levels of 16 other metabolites, serine, homoserine, threonine, fructose, rahmnose, NA1, NA3, glycerol, myo-inositol, glyceric acid, erythronic acid, galactonic acid, pyroglutamic acid, phosphoric acid, ethanolamine, and C18:3, were higher than at least one of the other stages (Supplemental Table S1).

Among the 19 amino acids detected, leucine, serine, aspartate, glutamate, asparagine, and glutamine (the last two are amides that have an additional nitrogen molecule) were found at the highest levels, above 100 nmol/g DW during at least one developmental stage (Figure 3; Supplemental Table S1). Since most of the soluble amino acids were incorporated into proteins (Cohen et al., 2014), we next studied if the compositions of the protein-incorporated amino acids that changed during the developmental stages. We tested the albumin fraction, which is the aqueous-soluble protein, representing about 80% of leaf proteins (Galili and Hofgen, 2002). The levels of isoleucine, valine, and aspartate increased significantly throughout the development, having the highest levels in mature plants (Table 1).

**Table 1:** **The levels of total amino acids in the four developmental stages of ***Phelipanche aegyptiaca*** after protein hydrolysis of the soluble proteins fraction, given in mol%^#^**.

**Protein-bound amino acids (%)**	**Tubercle**	**Pre-emergent shoot**	**Post-emergent shoot**	**Mature**
Glutamate	3.23 ± 0.31(*a*)	2.31 ± 0.22(*a*)	3.1 ± 0.14(*a*)	2.87 ± 0.22(*a*)
Aspartate	25.86 ± 1.5(*a*)	24.66 ± 1.24(*a*)	27.8 ± 0.95(*a*)	31.37 ± 0.28(*a*)
Lysine	1.48 ± 0.14(*a*)	2 ± 0.18(*a*)	0.94 ± 0.05(*a*)	1.24 ± 0.09(*a*)
Methionine	1.33 ± 0.08(*a*)	1.85 ± 0.09(*a*)	1.67 ± 0.08(*a*)	1.5 ± 0.08(*a*)
Threonine	5.23 ± 0.45(*a*)	4.94 ± 0.3(*a*)	4.44 ± 0.11(*a*)	4.21 ± 0.2(*a*)
Isoleucine	2.52 ± 0.2(*b*)	2.56 ± 0.16(*b*)	3.7 ± 0.14(*ab*)	5.21 ± 0.22(*a*)
Leucine	7.37 ± 0.44(*a*)	8.97 ± 0.53(*a*)	11.25 ± 0.44(*a*)	12.78 ± 0.25(*a*)
Valine	5.89 ± 0.26(*b*)	5.29 ± 0.23(*b*)	7.54 ± 0.31(*ab*)	10.05 ± 0.33(*a*)
Phenylalanine	10.9 ± 0.75(*a*)	8.14 ± 0.57(*a*)	8.31 ± 0.32(*a*)	8.7 ± 0.92(*a*)
Tyrosine	4.29 ± 0.34(*a*)	3.46 ± 0.29(*a*)	4.3 ± 0.54(*a*)	1.78 ± 0.2(*a*)
Glycine	10.37 ± 0.8(*a*)	8.05 ± 0.58(*a*)	10.72 ± 0.64(*a*)	9.84 ± 0.66(*a*)
Serine	7.77 ± 0.67(*a*)	8.24 ± 0.63(*a*)	7.39 ± 0.26(*a*)	7.53 ± 0.35(*a*)
Proline	1.68 ± 0.15(*a*)	1.89 ± 0.2(*a*)	1.94 ± 0.09(*a*)	2.92 ± 0.09(*a*)

# *The levels of amino acids were measured using GC-MS. The peak area was normalized to norleucine internal standard. For each biological replicate, the percentage (%) was calculated from the total of all amino acids that were detected at the specific sample, and then the average was calculated for all five biological replicates. Values are the mean ± standard deviation of five biological replicates, each taken from at least five plants. Significance was calculated according to the Turkey-Kramer HSD test (p < 0.05) and identified by different letters*.

We also studied fatty acid composition using another GC-MS method (Bai et al., 2012). The analysis detected six fatty, three saturated and three unsaturated acids (Supplemental Table S1). Except for α-linolenic (C18:3), whose levels significantly decreased from the pre-emergent shoot to the mature flowering stage (Figure 3), the levels of the other fatty acids did not change significantly between the four developmental stages. Notably, although the levels of most of the metabolites detected were relatively lower in the tubercle stage, the level of fatty acids did not decrease at this stage compared to the other developmental stages (Supplemental Table S1).

### Metabolic profile of five different *P. aegyptiaca* organs taken from mature flowering plants

**Figure 4:**
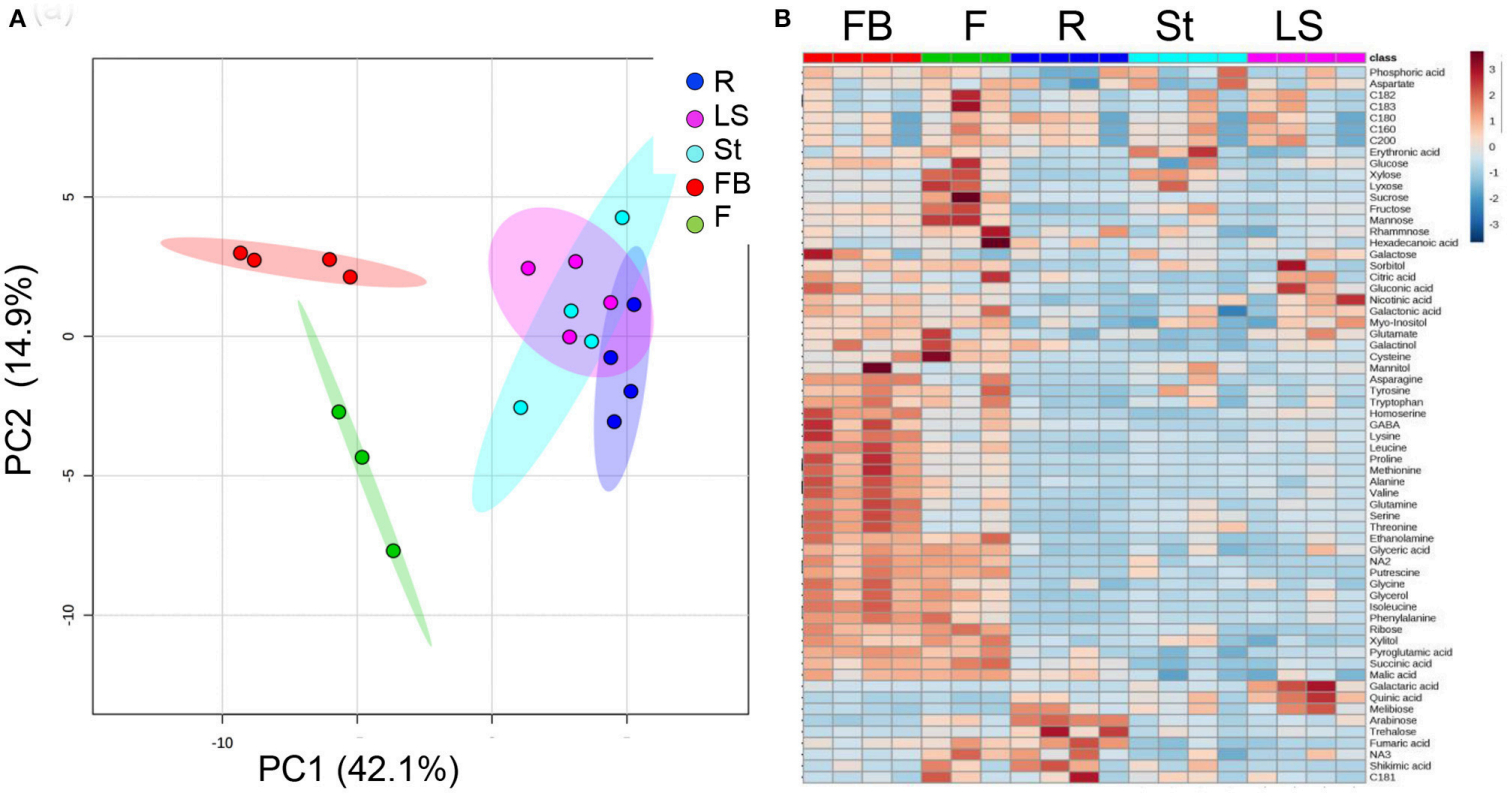
**Graphical representation of changes in metabolite profiles of five organs of mature ***Phelipanche aegyptiaca***. (A)** Principal component analyses (PCA) applied to the five organs according to their entire primary metabolome set of 66 metabolites. The data points are displayed as projections onto the two primary axes. Variances explained by the first two components (PC1 and PC2) appear in parentheses; **(B)** heat-map of these 66 primary metabolites detected by GC-MS. The data represent three to four replicates for each organ. The different organs are: Rt, adventitious roots; LS, underground pre-emergent shoot; St, post-emergent shoot; FB, Floral buds; and Fwr, Flowers.

The metabolic profiling of five different organs of the mature parasite was determined: Adventitious roots; underground pre-emergent shoot; post-emergent shoot; floral buds; and flowers (Figure 1). The metabolic profiles of these five organs were plotted onto a PCA to reveal that a separation exists between the three vegetative tissues, adventitious roots, pre- and post-emergent shoots to the reproductive organs, floral buds and flowers (Figure 4A). A heat-map analysis indeed determined that the two reproductive tissues (buds and flowers) have higher levels of most of the metabolites detected than the three vegetative organs (Figure 4B).

**Figure 5:**
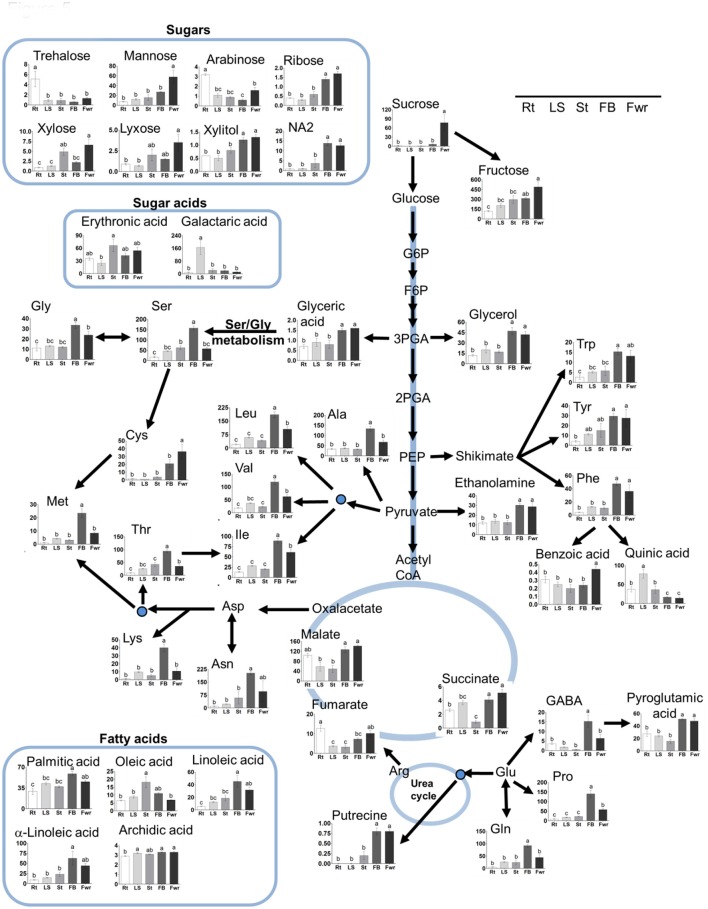
**The levels of primary metabolites and lipids of ***Phelipanche aegyptiaca*** in five organs of mature plants**. Only metabolites whose levels changed significantly in at least one developmental stage were presented. The different organs are: Rt, adventitious roots; LS, underground pre-emergent shoot; St, post-emergent shoot; FB, Floral buds; and Fwr, Flowers. The y axis of amino acids is shown in nmol/g DW, for fatty acid in μg/g DW, while for the other metabolites it represents the area of relative m/z response of each metabolite following normalization to the norleucine internal standard. Data shown are means ± *SE* of three to four replicates for each type of plant's organ. Significance was calculated according to the Turkey-Kramer HSD test (*p* < 0.05) and is identified by different small letters. For abbreviations of metabolite names, see Figure 3, except for abbreviations of Asn, asparagine; Gly, glycine; Tyr, tyrosine; Phe, phenylalanine; Ala, alanine; Lys, lysine; Gln, glutamine.

To gain a better resolution, each metabolite whose level changed significantly between the different organs was plotted separately on Figure 5. The results showed that the levels of the 15 soluble amino acids increased significantly in the floral buds compared to the three vegetative organs. The levels of 11 of them decreased significantly in the flowers (Figure 5; Supplemental Table S2). In addition, the levels of non-protein amino acids (homoserine and GABA) increased significantly in the floral buds. The levels of asparagine and the three aromatic amino acids (tyrosine, phenylalanine, and tryptophan) displayed similar high levels in both flowers and buds, while the level of cysteine was found to be higher in flowers. Notably, the levels of aspartate and glutamate did not differ significantly between the five organs, while those of asparagine and glutamine, which show relatively high levels, accumulated preferentially in the reproductive organs (Figure 5; Supplemental Table S2). The amino acids whose levels were the highest (over 100 nmol/g DW) in the floral buds were leucine, valine, serine, asparagine, glutamate, threonine, alanine, and proline.

The higher levels of soluble amino acids in the floral buds might be the result of lesser incorporation of these amino acids into proteins. To test this possibility, we studied the compositions of the protein-incorporated amino acids in the albumin fraction of the different organs. The analysis shows that five out the 13 amino acids changed significantly between the organs. Adventitious roots had higher levels (by means of mol%) of phenylalanine and tyrosine compared to the flowers, while the floral buds had significantly higher levels of the three branch chain amino acids, isoleucine, leucine, and valine, compared to the other organs (Table 2). Therefore, the results suggest that the total levels of branch chain amino acids (soluble and protein-incorporated) increased in the floral buds. The higher levels of other soluble amino acids did not significantly affect their incorporation to the proteins.

**Table 2:** **The levels of total amino acids in five organs of mature flowering plants of ***Phelipanche aegyptiaca*** after protein hydrolysis of the soluble proteins fraction, given in mol%^#^**.

**Protein-bound amino acids (%)**	**Adventitious roots**	**Lower shoot**	**Stem**	**Floral buds**	**Flowers**
Glutamate	4.68 ± 0.45(*a*)	4.44 ± 0.37(*a*)	2.88 ± 0.25(*a*)	1.15 ± 0.22(*a*)	2.16 ± 0.55(*a*)
Aspartate	22.71 ± 1.13(*a*)	18.92 ± 1.18(*a*)	22.52 ± 1.26(*a*)	16.94 ± 0.3(*a*)	23.31 ± 0.7(*a*)
Lysine	2.31 ± 0.83(*a*)	1.16 ± 0.08(*a*)	0.82 ± 0.04(*a*)	1.77 ± 0.09(*a*)	1.84 ± 0.13(*a*)
Methionine	1.76 ± 0.15(*b*)	3.08 ± 0.12(*a*)	1.59 ± 0.14(*b*)	2.42 ± 0.15(*ab*)	1.7 ± 0.12(*b*)
Threonine	3.84 ± 0.08(*a*)	5.07 ± 0.19(*a*)	4.34 ± 0.34(*a*)	6.25 ± 0.52(*a*)	5.26 ± 0.32(*a*)
Isoleucine	3.59 ± 0.08(*b*)	4.99 ± 0.13(*b*)	4.73 ± 0.3(*b*)	8.49 ± 0.21(*a*)	8.1 ± 0.25(*a*)
Leucine	7.64 ± 0.35(*c*)	10.7 ± 0.26(*b*)	9.32 ± 0.6(*bc*)	15.38 ± 0.23(*a*)	13.7 ± 0.23(*ab*)
Valine	8.76 ± 0.48(*b*)	10.6 ± 0.42(*b*)	10.25 ± 0.64(*b*)	17.2 ± 0.44(*a*)	12.4 ± 0.68(*ab*)
Phenylalanine	15.84 ± 0.44(*a*)	12.9 ± 1.08(*ab*)	11.3 ± 1.18(*ab*)	5 ± 0.51(*b*)	6 ± 0.32(*b*)
Tyrosine	3.75 ± 0.33(*a*)	1.13 ± 0.15(*b*)	2.19 ± 0.19(*ab*)	2.14 ± 0.25(*ab*)	1.27 ± 0.13(*b*)
Glycine	13.56 ± 0.55(*a*)	13.72 ± 0.54(*a*)	9.14 ± 0.85(*a*)	11.58 ± 1.25(*a*)	11.97 ± 0.77(*a*)
Serine	7.77 ± 0.17(*a*)	9.63 ± 0.34(*a*)	8.53 ± 0.61(*a*)	8.47 ± 0.36(*a*)	8.78 ± 0.44(*a*)
Proline	3.79 ± 0.32(*a*)	2.77 ± 0.09(*a*)	2.62 ± 0.17(*a*)	3.22 ± 0.18(*a*)	3.44 ± 0.32(*a*)

# *The levels of amino acids were measured using GC-MS. The peak area was normalized to norleucine internal standard. For each biological replicate, the percentage (%) was calculated from the total of all amino acids that were detected at the specific sample, and then the average was calculated for all five biological replicates. Values are the mean ± standard deviation of five biological replicates, each taken from at least five plants. Significance was calculated according to the Turkey-Kramer HSD test (p < 0.05) and identified by different letters*.

In addition to amino acids, the levels of other 28 metabolites changed significantly in at least in one organ compared to the others. The levels of the sugars sucrose and mannose were significantly higher in the flowers, while fructose, ribose, and NA2 had higher levels in flowers as well as in floral buds (Table S2). Flowers and/or floral buds had higher levels of glycerol, xylitol, malic acid, succinic acid, glyceric acid, benzoic acid, pyroglutamic acid, putrescine, and ethanolamide. In total, 37 metabolites increased in one or both of these two reproductive organs. However, adventitious roots showed the highest levels of trehalose and arabinose, while the pre-emergent shoot had the highest levels of quininc and galactaric acids (Figure 5).

Next, we studied fatty acid composition. As shown for most of the other metabolites, floral buds have higher levels of three fatty acids, linoleic (C18:2), α-linolenic (C18:3), and palmitic (C16:0), compared to the previous stages. However, oleic acid (18:1) was higher in the pre-emergent shoot compared to the adventitious roots, post-emergent shoot, and flowers (Figure 5; Supplemental Table S2). The level of stearic acid (18:0) did not change significantly between the different organs.

### The contents of total nitrogen, total soluble protein in the albumin fraction, and total reducing sugars (represented as starch)

**Figure 6:**
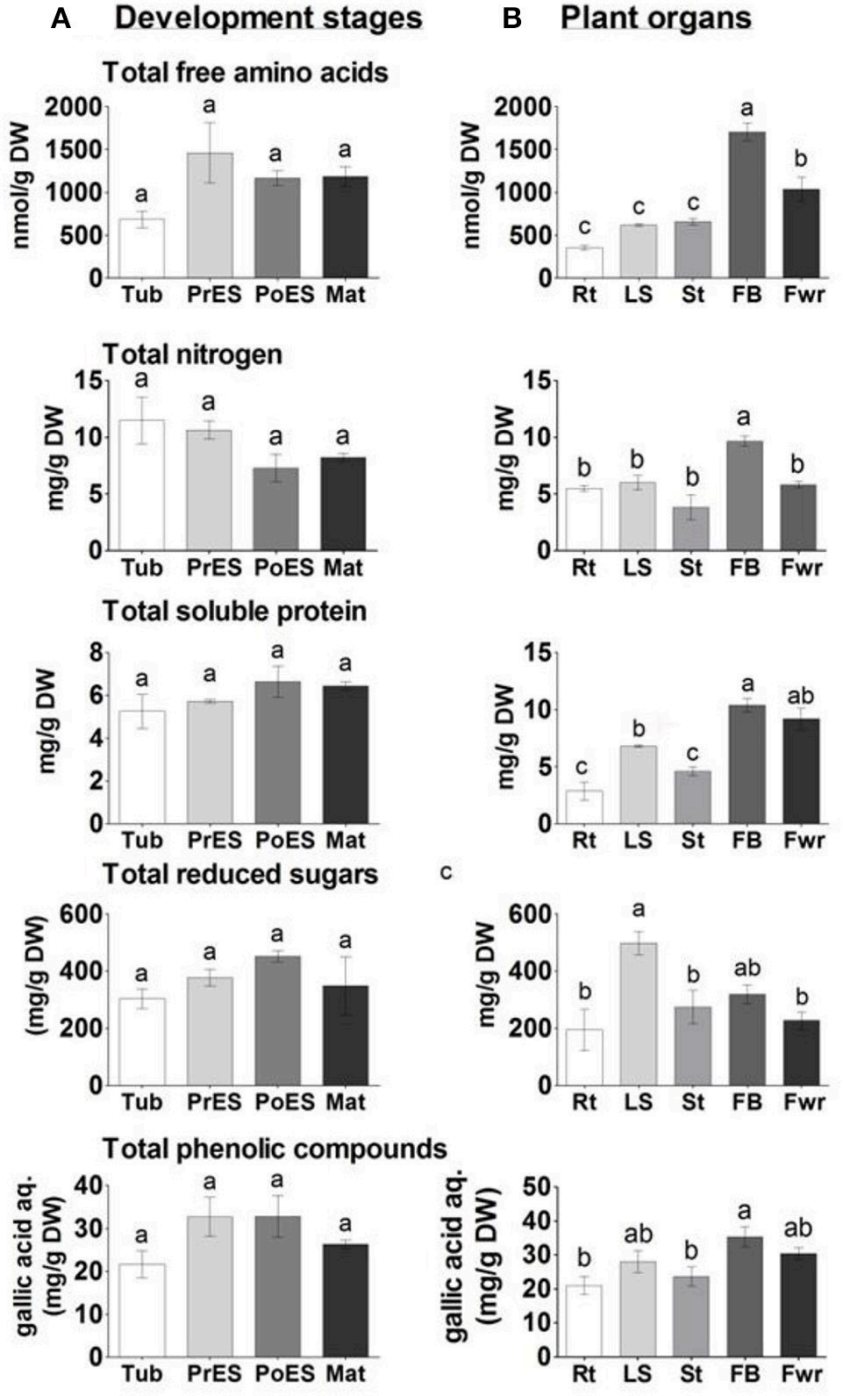
**The levels of total free amino acids, nitrogen, soluble proteins, reduced sugars and total phenols in ***Phelipanche aegyptiaca*** at four developmental stages (A)**; and in five organs from mature plants **(B)**. Total free amino acids contents measured by GC-MS analysis; total protein contents in the albumin fraction as measured using a Bradford assay; total reducing sugar contents analyzed after hydrolysis by the Sumner method; and total polyphenol content (represented as mg gallic acid equivalents per L of water extract). Data shown are means ± *SE* of three to four replicates for each sample. Significance between plants was calculated according to the Turkey-Kramer HSD test (*p* < 0.05) and is identified by different small letters. Tub, Tubercle; PeES, Pre-emergent shoot; PoES, Post-emergent shoot; Mat, Mature; Rt, adventitious roots; LS, underground pre-emergent shoot; St, post-emergent shoot; FB, Floral buds; and Fwr, Flowers.

The higher levels of 11 out of the 18 amino acids in the last three developmental stages, and the relatively higher levels of the total amino acids (Figure 6A), encouraged us to determine the total nitrogen content in these stages. The analyses revealed that there were no significant differences between the different developmental stages (Figure 6A). Next, the total soluble proteins (albumin fraction) were examined using the Bradford analysis. The analyses again revealed that there were no significant differences between the different developmental stages (Figure 6A).

The levels of total soluble amino acids, total nitrogen, and soluble proteins were also examined in the five organs of the adult plants. Floral buds had significantly higher levels of total free amino acids and nitrogen compared to other organs (Figure 6B), while soluble proteins were higher in floral buds and flowers. This indicates that the reproductive organs, especially the floral buds, have higher levels of free amino acids, nitrogen, and soluble proteins. Adventitious roots, however, had about 5-, 2-, and 4-fold lower levels of total soluble amino acids, nitrogen and soluble proteins, respectively, than the floral buds.

Changes are also expected due to the finding that the levels of simple sugars changed significantly during the development and in the organs tested. To this end, we examined the levels of total reducing sugars (represented as starch) following carbohydrate hydrolysis. The reducing sugars did not change significantly during the developmental stages. However, their levels increased significantly in the underground pre-emergent shoot by 2.7-fold compared to the adventitious roots and flowers (Figure 6B).

### Determination of total soluble phenolic compounds

We previously determined that *P. aegyptiaca* has about a three-fold higher level of total phenols (most of them secondary metabolites) compared to the host's roots (Hacham et al., 2016). Determination of total phenols content showed that their levels did not change significantly in the four developmental stages (Figure 6A). In plant organs, however, the floral buds had 45 and 34% more phenols than the adventitious roots and post-emergent shoot, respectively (Figure 6B).

## Discussion

### Differences in metabolic profiling during the four developmental stages

Determination of the metabolic profile of the four developmental stages revealed that the levels of 35 out of the 66 metabolites, which come from all determined biochemical groups, differed significantly in at least one of the developmental stages. However, the levels of 31 metabolites (constituting 45% of the total) still did not change significantly during development (Supplemental Table S1). This finding is unlike those reported for various developmental stages in different organs of non-parasitic plants. In the latter plants, the levels of most of the detected metabolites changed significantly during development. For example, significant changes were found in the levels of soluble and protein-bound amino acids during the development of all Arabidopsis aerial organs (rosette leaves, upper leaves, flowers, siliques, seeds) (Frank et al., 2015). In addition, the levels of all metabolites examined in the primary metabolic profiling of Arabidopsis, sunflower, and tobacco leaves changed significantly during their developmental stages (Watanabe et al., 2013; Li et al., 2016; Moschen et al., 2016), and also in the upper leaves and siliques in Arabidopsis (Watanabe et al., 2013). Although we compared between the whole *P. aegyptiaca* plants to the organs in non-parasitic plants, we assume that the metabolic behavior of the parasite is considerably less altered during development compared to non-parasitic plants. We hypothesize that the gap between *P. aegyptiaca* and non-parasitic plants in the levels of the different metabolites is one of the significant differences between the two types of plants. Although the reasons for such changes are not yet known, we assume that it is related to the life cycle of these two types of plants. For example, the leaves of non-parasitic plants served as a sink when they were young, as a source when they matured, and at senescence, most of their metabolites transferred toward the developing reproductive tissues including seeds. These led to significant changes in their metabolic profiling during development (Gregersen and Holm, 2007; Lim et al., 2007; Gregersen et al., 2008; Watanabe et al., 2013; Distelfeld et al., 2014; Moschen et al., 2016). However, *P. aegyptiaca*, as a parasitic plant, does not have functional leaves, and as a parasite, it relies at least for its carbon source on its hosts. This might be the reason for lesser change in the levels of their metabolites. The results also proposed that the parasite can control the levels of about half of its metabolites during its developmental stages.

Basically, the tubercle stage tends to have the lowest average level of amino acids, sugars and other metabolites compared to the other stages (Figures 2B, 3). This suggests that the parasite at this stage attracts metabolites relatively less from the host, or has a lesser ability to produce many of these metabolites compared to the more advanced developmental stages. Among the metabolites identified, only trehalose was significantly higher at the tubercle stage compared to the other stages. The level of trehalose increased significantly in *P. aegyptiaca* infected tomato roots compared to the non-infected roots (Hacham et al., 2016). Trehalose is known to be involved in stress responses, plant-microorganism interactions and defense response against pathogens (Zhang et al., 2016). Therefore, it might be that the significantly higher level of trehalose in the tubercle stage resulted from the infected roots that had higher levels of this metabolite that transferred to the parasite. Despite this assumption, we cannot exclude the possibility that they also form in the parasite.

The second stage (pre-emergent shoot) had the highest total hydrophilic metabolites (55% more than the tubercle and 33% more than in the two later developmental stages; Supplemental Table S1). This implies that the pre-emergent shoot became a much stronger sink than the tubercle, gained more abilities to synthesize these metabolites, and/or utilized these metabolites less. However, the levels of all of these metabolites tend to decrease in the last two developmental stages when the reproductive organs are produced (Figure 3). This implies that they are used as precursors for the synthesis of other metabolites, and/or as respiratory and energy substrates. We also assume that the plant at this pre-emergent stage accumulates metabolites to support emergence from the ground when relatively rapid growth occurred.

### Differences in metabolic profiling in the five different organs of the mature plant

Non-parasitic plants exhibit significant differences in their primary metabolites between different organs during development (Watanabe et al., 2013; Frank et al., 2015). However, only 44 metabolites out of 66 changed significantly between the different organs of the mature *P. aegyptiaca* plants (Supplemental Table S2). Yet, 22 metabolites (representing 32% of the total) were not significantly changed, suggesting that the parasite has the ability to control the levels of these unchanged metabolites in its organs.

PCA and heat-map analyses of the organs of the mature parasite showed a distinguished separation between the vegetative and reproductive organs. Indeed, it was observed that floral buds and flowers have the highest levels of most metabolites compared to vegetative organs (29 in buds and 19 in flowers). The gap in levels between floral buds and flowers proposed that several metabolites were used in the flowers to synthesize other metabolites that cannot be detected by the methods used in the current study, or they were used as an energy source, as proposed from the higher levels of malate and succinate in this organ (Figure 5). These results are similar to those reported for non-parasitic plants that exhibit diverse primary metabolic profiling in their different organs during development (Gregersen and Holm, 2007; Watanabe et al., 2013; Distelfeld et al., 2014). The major differences in the metabolom is largely shown during the transition between the vegetative to the reproductive stage (Gregersen et al., 2008; Watanabe et al., 2013; Li et al., 2016; Moschen et al., 2016). This metabolic shift is a key for the mobilization and recycling of nutrients from senescing leaves to sinks, such as young leaves, storage tissues, and/or developing seeds (Lim et al., 2007).

Out of the 16 soluble amino acids whose levels changed significantly in reproductive organs, 12 had the highest contents in floral buds (Figure 5; Supplemental Table S2). The total soluble amino acids in this organ were 4.8-fold higher than in adventitious roots, which exhibited the lowest accumulation (Figure 6B). Relatively higher levels were also detected in flowers. These elevations were in accordance with a higher level of soluble proteins, and higher total nitrogen content in these organs, especially in the floral buds (Figure 6B), which are required to prepare the flowers and the internal reproductive tissues. Additionally, higher levels of ethanolamine, putrescine, GABA, and pyroglutamic acid were detected in buds and/or flowers (Figure 5; Supplemental Table S2). This suggests that these organs required further nitrogen compounds (in addition to amino acids), since all of them have an amine group. They are proposed to contribute to the high levels of nitrogen, at least in the buds. However, they might also play a role in flowers, since putrescine synthesis is correlated metabolically during flower development in Citrus and Polianthes, and its changes are involved in floral initiation and development (Huang et al., 2004; Zierer et al., 2016). As suggested for non-parasitic plants, the higher soluble compounds in the reproductive organs decreased the water potential and attracted more metabolites toward this organ from the other organs, which were then used for flower and seed production.

The levels of three soluble aromatic amino acids increased in the flower buds compared to the other organs (Figure 5). In addition, the levels of phenylalanine decreased in the buds and flowers in the protein fraction. These two observations proposed that the pathways derived from phenylalanine (such as phenylprepnoinds) were enhanced in these two organs, which apparently contribute to the color (anthocyanins) and volatiles (like benzenoids) of flowers. Indeed, the high level of benzoic acid in flowers proposed that it is used for the synthesis of volatile benzenoids that are known to attract pollinators (Khurana and Cleland, 1992). This reduction of phenylalanine in the protein fraction is also apparently related to the significantly higher levels of total phenols found in the buds (Figure 6). Higher levels of aromatic amino acids and total phenols (about 3-fold) compared to the host's roots were previously detected in tubercle (Hacham et al., 2016), suggesting that these pathways are active in the parasite.

In addition to these compounds, flowers exhibit high levels of sucrose and mannose, while fructose, ribose and NA2 have higher levels in flowers and in floral buds (Supplemental Table S2). The level of sucrose, which was relatively very low in the vegetative organs of the parasite, increased in flowers by about 3.3-fold compared to the adventitious roots. Sucrose is proposed to be one of the main carbohydrates that are channeled from the host toward the *O. crenata* (Aber et al., 1983). However, the sucrose level in *P. aegyptiaca* was 77% lower than in the infected tomato roots, while the level of its two components, glucose and fructose, increased significantly in the parasite (Hacham et al., 2016). This is mainly due to the invertase activity that was indeed detected in *P. ramosa* (Draie et al., 2011). We assume that the high levels of sucrose and hexoses in flowers are due to the presence of nectar, which are the predominant floral nectar sugar in many species (Wolff, 2006).

Although most of the metabolites, including the total proteins, nitrogen, and phenols are accumulated in the reproductive organs, the levels of the reducing sugars representing starch increased significantly in the underground pre-emergent shoot (Figure 6B). The accumulation of reducing sugars proposes that this organ serves as a reservoir of carbohydrates for the upper organs. One possibility is that this reservoir can be used when the upper shoot is removed, which enables the synthesis of other shoots, an assumption that should be tested, and/or when the sullies from the host are reduced. We also assume that the less soluble sugars in this organ (Figure 5) enable the water coming from the host to be channeled through this organ to the upper organs at the mature stage.

This study provides more knowledge about changes occurring in the metabolic profiling during the developmental stages and in five organs of mature *P. aegyptiaca* plants. In the future, it would be worthwhile to reveal which metabolites are derived from the host, and which are mainly synthesized inside the parasite. Such data could contribute to our knowledge about the behavior of *P. aegyptiaca* and its metabolic nature. Differences in the metabolic pathways and their regulation between *P. aegyptiaca* and its hosts could enable additional approaches in the future to control this parasite.

## Author contributions

YH and RA conceived and designed the research. NN and YH conducted all of the experiments and data analyses. JH and ED performed the growth and collection of the parasite, and assisted in the design part of the analyses. RA and YH prepared the manuscript. All authors read and approved the manuscript.

## Funding

This work was supported by a grant from the JCA Foundation (Accelerator project).

### Conflict of interest statement

The authors declare that the research was conducted in the absence of any commercial or financial relationships that could be construed as a potential conflict of interest.

## Abbreviations

GC-MS: Gas chromatography-mass spectrometry
GABA, γ-aminobutyric acid TCA: tricarboxylic acid cycle
PCA: principal component analysis.
